# Supplementary material for: Antimicrobial-Resistant Bacteria from Free-Living Green Turtles (Chelonia mydas)
Source: Antibiotics (Basel). 2023 Aug 1;12(8):1268. doi: 10.3390/antibiotics12081268 (PMC10451770; doi:10.3390/antibiotics12081268)

Table S1: Species identified using MALDI-TOF and 16S rRNA Sanger sequencing.

Species	Number of isolates	MALDI-TOF identification: N (score)	16S rRNA N (bp /% Identity)
**Neck**			
C. freundii	2	2 (2.262)	_
E. coli	1	1 (1.835)	_
Salmonella sp.	1	1 (1.842)	_
S. ureylitica	1	1 (2.281)	_
P. mirabilis	1	1 (2.010)	_
E. faecalis	8	6 (2.255)	2 (504/97)
Microbacterium spp.	5	_	5 (423/98)
C. cellulans	8	5 (2.035)	3 (504/99)
**Cloaca**			
Species	Number of isolates	MALDI-TOF identification N (score)	16S rRNA N (bp /% Identity)
C. braaki	2	2 (2.043)	_
C. freundii	20	20 (2.281)	_
K. oxytoca	1	1 (2.202)	_
K. variicola	1	1 (1.887)	_
Salmonella sp.	1	1 (1.771)	_
S. marcescens	1	1 (2.405)	_
M. morganii	40	39 (2.180)	1 (446/98)
P. mirabilis	14	14 (2.277)	_
E. faecalis	9	7 (2.199)	2 (516/94)
E. hirae	2	1 (2.464)	1 (390/98)
L. garvieae	1	1 (2.098)	_

N, number of isolates; score, score values; bp, base pair; % Identity of the query with reference

Table S2. Metadata of the 16 captured green turtles.

Turtles	Left Fin Tag	Right Fin Tag	Weight (kg)	CCL (cm)	CCW (cm)	Samples*
C. mydas 1	BR84264	BR85265	96.6	92.0	80.5	1C/1N
C. mydas 2	BRA18245	BR97696	30.9	56.1	63.2	2C/2N
C. mydas 3	BRA18260	BRA18259	74.5	71.6	82.1	3C/3N
C. mydas 4	BRA18216	BRA18215	22.5	49.9	56.8	4C/4N
C. mydas 5	BR97680	BR97679	32.9	64.0	64.2	5C/5N
C. mydas 6	BR97657	BR97689	42.5	66.8	59.5	6C/6N
C. mydas 7	BRA17672	BRA18262	31.8	54.4	62.1	7C/7N
C. mydas 8	BR97684	BR97683	36.5	64.0	55.7	8C/8N
C. mydas 9	BR84237	BR84238	70.0	73.9	74.7	9C/9N
C. mydas 10	BRA18254	BRA18253	28.0	42.8	40.4	10C/10N
C. mydas 11	BRA18214	BRA18212	16.9	49.2	43.9	11C/11N
C. mydas 12	BR97674	BR97673	28.5	50.4	59.5	12C/12N
C. mydas 13	BRA18237	BRA18238	40.3	65.0	69.4	13C/13N
C. mydas 14	BRA18220	BRA18219	12.6	49.3	50.5	14C/14N
C. mydas 15	BR97667	BRA18232	40.9	61.6	69.7	15C/15N
C. mydas 16	BR97678	BRA18218	28.4	60.4	51.4	16C/16N
		Average	39.6	60.7	61.5	
		Median	32.3	61.0	60.8	
		Stdev	22.4	12.2	11.9	

*C - cloaca, N - neck

Figure S1: Map of study location. **A**, Map of South America, showing Brazilian coast, highlighting Rio de Janeiro state littoral; **B,** Rio de Janeiro state shoreline, highlighting Guanabara Bay; **C**, Guanabara Bay, highlighting (red dot) Itaipú Beach, where the green turtles were captured.

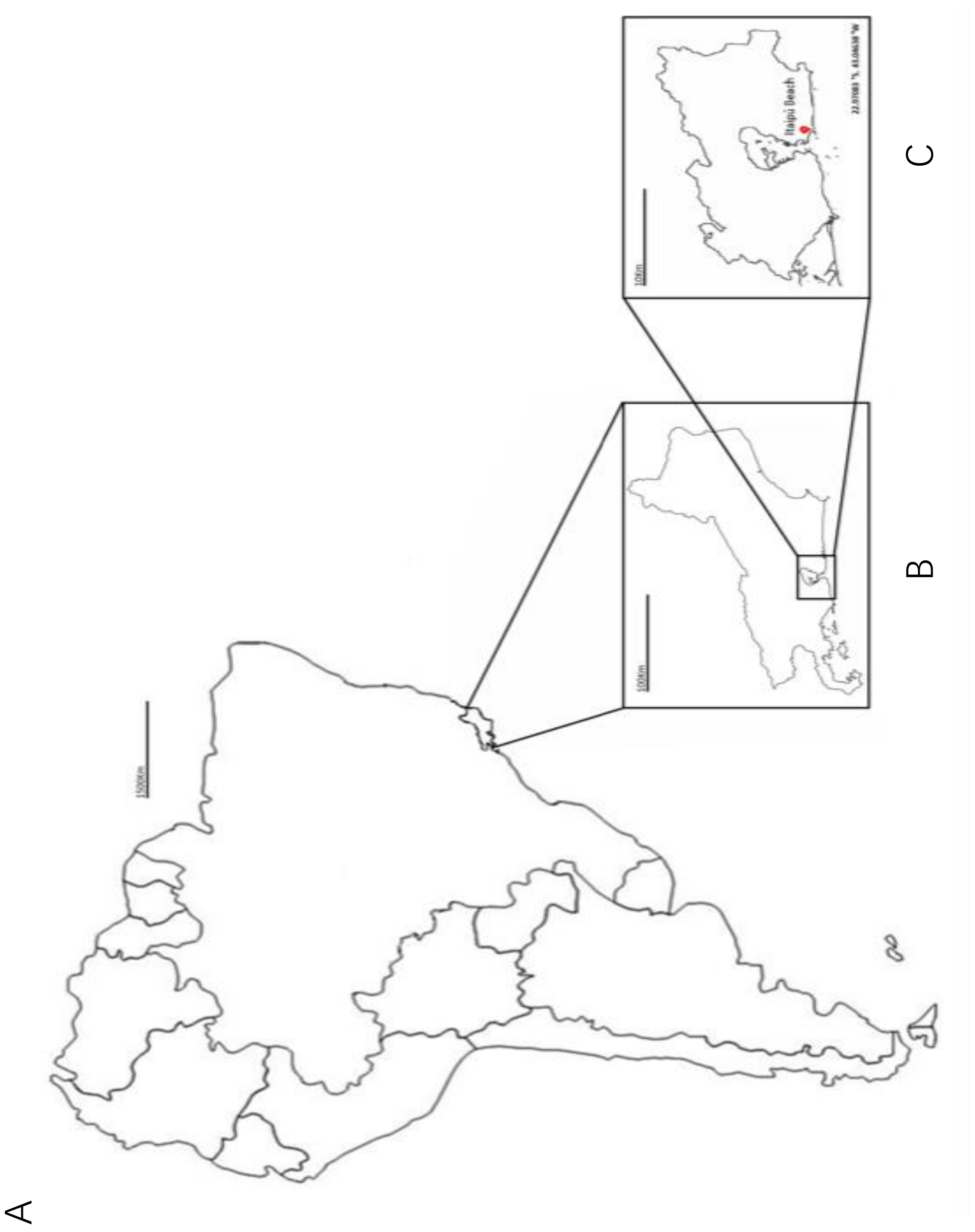

Supplement: Supplementary file 1 [file antibiotics-12-01268-s001.zip › antibiotics-2506432-supplementary.pdf]
